# Supplementary material for: Effect of pH on Adsorption of Tetracycline Antibiotics on Graphene Oxide
Source: Int J Environ Res Public Health. 2023 Jan 30;20(3):2448. doi: 10.3390/ijerph20032448 (PMC9915905; doi:10.3390/ijerph20032448)
Supplement: Supplementary file 1 [file ijerph-20-02448-s001.zip › ijerph-2155803-supplementary.pdf]

Table S1. The regression equations of TC、OTC, and CTC.

Antibiotics	Regression equation	R^2	Linear range（mg/L）
Tetracycline hydrochloride（TC）	A=0.0288x-0.0042	0.9993	0-50
Oxytetracycline hydrochloride（OTC）	A=0.0294x-0.0025	0.9984	0-50
Chlortetracycline hydrochloride（CTC）	A=0.0268x-0.0028	0.9988	0-50

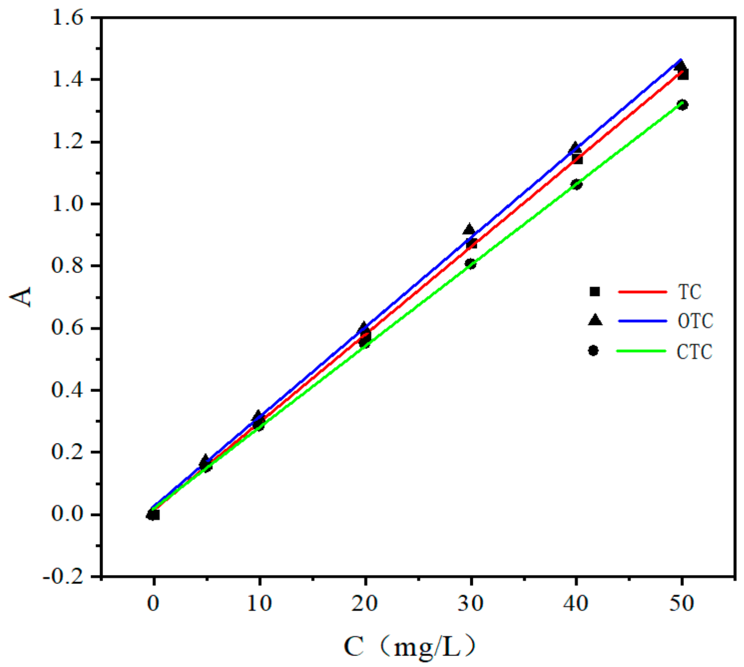

Figure S1. The calibration curves of TC, OTC, and CTC.

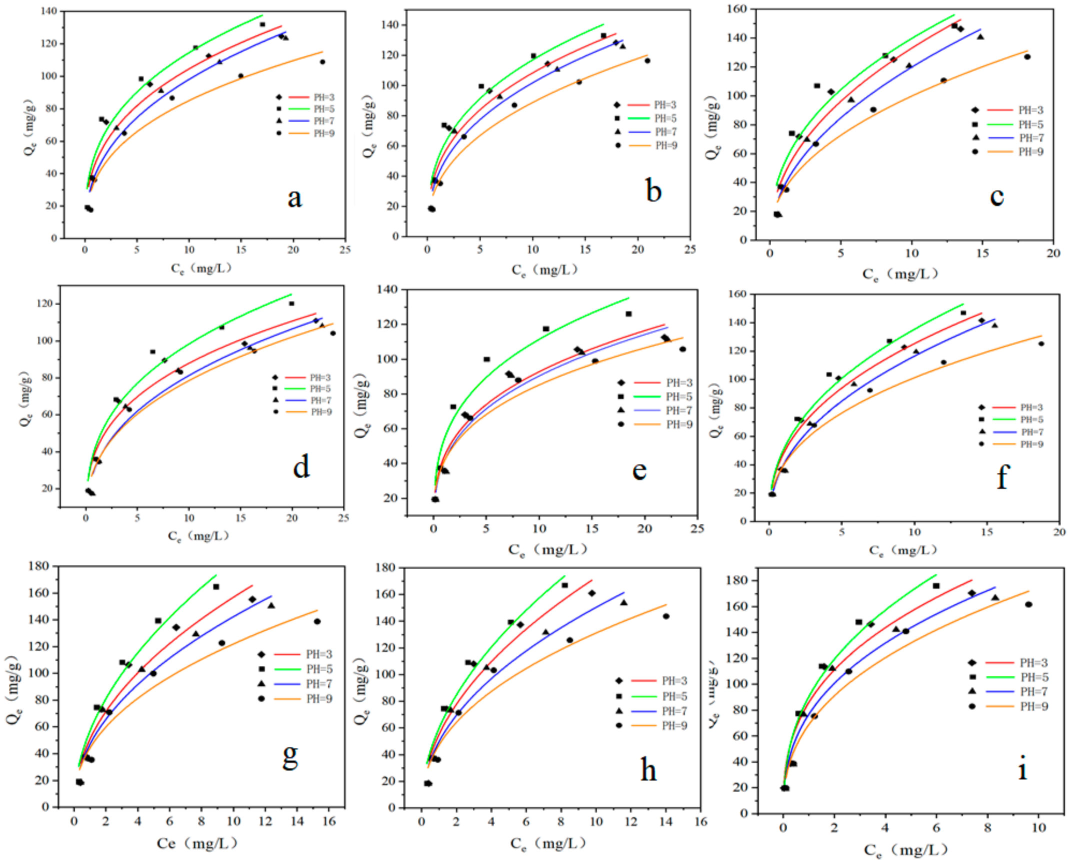

Figure S2. Freundlich adsorption isotherm model (a, b, c are the adsorption of GO-TC at 288 K, 298 K, 308 K; d, e, f are the adsorption of GO-OTC at 288 K, 298 K, 308 K; g, h, i are the adsorption of GO-CTC at 288 K, 298 K, 308 K, respectively).
